# Scotch Physicians and Surgeons

**Published:** 1888-04-21

**Authors:** C. G. Furley


### Scotch Physicians and Surgeons.

By C. G. Furley.

*(Continued from p. 7.)*

### JAMES SYME.

There are two names which go together in the history of surgery in Edinburgh—Liston and Syme—and if we here put the second name before the first, and tell Syme's story before touching on Liston's, it is because though he was by a few years the younger man his lifelong practice in Edinburgh associates his name more closely than that of his senior with the medical school of the city.

James Syme was born in Edinburgh, at No. 56, Princes Street, on the 7th November, 1799. The house is no longer standing, having been pulled down to make way for an enlargement of the Royal Hotel; but knowing its site, one can still stand and survey the scene—one of the most beautiful even in beautiful Edinburgh—on which his eyes first looked. First, a gay and busy street, more gay and busy now than it was ninety years ago, when shops were less continuous and private houses more common; but from its first days the favourite promenade of all in "the grey metropolis of the north" who love gaiety and sunshine. On the other side of the street the eye falls on that Gothic pyramid, the Scott monument. There was, of course, no Scott monument in 1799, for Mr. Scott, the rather lazy lawyer and good-natured sheriff, with more knowledge of old ballads and legends than of "multiplepoinding" and other mysteries of Scots law, had not yet become the "great unknown," nor blossomed into "Sir Walter." The steep valley beyond, now a garden whose amenity is somewhat marred by the incursion of the railway, had then neither rails nor flower-beds, but was a lake—not to hint the word swamp—from whose further shore rose the tall houses of the old town, and the Castle Rock, precipitous and grey, though softened by the stray plants of wallflower and ivy that cling to its crevices, crowned with the ancient battlements that were placed there for more practical ends than mere picturesqueness of effect, however well they serve that purpose now.

The Castle Rock should—we cannot assuredly say it did, much as we should like to make the truth and the fitness of things join hands—but it should have exercised a strong fascination over James Syme's childish fancy. He was in many ways as hard and unyielding as the rock, and like those who built the castle he was "ever a fighter." The record of his life is a record of struggle, and, unfortunately, of quarrels.

He was not malicious, he was not irritable; but he was *dour*—no English word can quite express that quiet immovability, that determination to go on in a way once chosen, without deviation for love of friend or fear of foe, which the Franco-Scottish monosyllable implies. *Dour* men are just, but hardly generous; they receive admiration, respect, obedience, oftener than love; they make friends by dint of their undoubted merit, but seldom win them by that subtle sympathy of soul which is perhaps a surer, if less palpable claim.

He could not help his obstinacy; it came by inheritance from a father who by means of it dissipated almost all other. John Syme, the surgeon's father, was, to begin with, a prosperous man, a lawyer by profession—to be strictly accurate, a writer to the Signet—and the owner of an estate in Kinross-shire and one in Fife. But he had a fancy for speculating in land whose chief value consisted in minerals supposed—with or without reason—to be lying beneath the surface. Experimental bargains of this sort—dips into the lucky-bag of mother earth—are lotteries of a very risky sort; and the end of John Syme's purchases of land was his being forced to sell his family estate of Lochore, in Fife. He might have ended his days as a richer man if he could have been persuaded to give up his speculations when first they turned out unfortunately; but he clung to them persistently. To do otherwise would have been giving in, and he would not give in. Neither would his son after him.

James Syme went through the usual educational course of Edinburgh boys in the early part of the century. After a few years at a preparatory school, he went to the High School, where in a class of one hundred and thirty he took the respectable but not brilliant place of twenty-fifth boy. He was not quick at his books; he never loved them; he gained knowledge by sheer hard work. When his school-days came to an end he startled his companions by a speech which, though a touch of the melodramatic may have been added in repetition, must, even in its original form, have been startling enough. While standing in the midst of a group of boys, talking of school events, the successes and failures of the past session, he suddenly threw his class-books from his arms, exclaiming: "Away with these toys; I am now done with them; the more serious business of life is before me, and to it I mean to address myself."

These schooldays had not been notably happy ones for him. He suffered not only from a certain shyness of temperament which might easily pass for reserve or even sullenness, but from a thickness of speech which at first was almost an impediment. By dint of long-continued effort this at last nearly disappeared; but while he was at school it was very noticeable, and one of his masters insisted on assuming that it was affected for the purpose of shirking lessons, and acted severely on this hypothesis—a course which may have tended to develop Syme's dislike to schooling. But he was altogether, from a schoolboy's point of view, eccentric. He did not care for games—for football, or "shinty," or "dully," or "the papes," or any of the sports which delight Edinburgh children, and which Christison, even when on the graveward side of seventy, recalled with enthusiasm; he preferred—this friend and contemporary of Christison's—a quiet talk with the few friends he cared for, a walk in the fields with a view to learning the names and characters of the plants that grew there, or to catching small animals—frogs and lizards and field mice, which he subsequently reduced to skeletons with a dexterity and skill which in later days, among more experienced companions, was considered noteworthy and admirable. But the pursuit of natural science, except it be attended by possible injury to life and limb, and actual damage to skin and garments, is not attractive to the average schoolboy, so Syme's childhood was a lonely one, and he must have suffered to some extent the misery of being misunderstood.

These eccentric habits of his, along with a taste for chemical investigation, proved, probably, the impetus which led him to the study of medicine—the only recognised profession of those days which gave a man scope to study the natural sciences. He did not, however, advance far in the strict science of botany, but in later life he took to horticulture with no little success. In his earlier student days the zoological studies also fell into the background, while he took up chemistry with enthusiasm. He was a member of that chemical society whose feats and misadventures are narrated by Christison, and not content with the knowledge gained at its weekly meetings, he pursued investigations by himself, the result being that he alone, of all who belonged to the society, distinguished himself by making a discovery.

It was rather a notable one for a lad of nineteen to chance upon, and brought a good deal of money to—not the inventor—but the patentee. On March 5th, 1818, he announced, in a short communication which he sent to the editor of the *Annals of Philosophy*, that he had discovered a new and cheap solvent for caoutchouc—namely, coal-tar naphtha. "As coal-tar," he said, "bears a strong resemblance to petroleum, it occurred to me that by distilling it a fluid might be procured which, like naphtha, would have the property of dissolving caoutchouc, and that in this way I could procure a solvent, free from the objections to which the known solvents of that remarkable substance are all more or less liable. After many trials I completely succeeded, and was enabled to carry into effect several of the applications for which a fluid state of india-rubber had seemed so desirable. Thus I constructed flexible tubes of the substance itself, and rendered various textures waterproof by brushing a thin solution of it into their interstices. A silk cloak, which afforded complete protection from the heaviest rain, and could also be used as a pitcher by turning up its skirt, was a wonder to all who saw it."

We owe, then, our useful "mackintoshes" to a surgeon, though they bear the name of the Glasgow manufacturer who, seeing Syme's account of his discovery in the *Annals of Philosophy* for August, 1818, secured the right of making cloth waterproof by this process. The discoverer's friends did indeed suggest his taking out a patent; but he declined to do so, because, as he says, "he was about to commence a profession with which considerations of trade in those days did not seem to be consistent."

The medical profession is not afraid now to base its claims to respect on wider grounds than refusing to touch money gained by honourable inventions. Narrow professionalism of that sort, drawing its learned skirts away from trade and saying to it, "I am worthier than thou," has had its day, like other "isms"; we esteem a man highly now by what he does, not by what he refrains from doing. And, knowing the years of struggling poverty which almost every doctor must accept who wants to do good work, and finally take a high place in his profession, knowing too that in his early years Syme suffered from serious pecuniary anxieties, we cannot help wishing that he had profited by his useful invention, and that we were therefore wearing "Symes" instead of "Mackintoshes" on rainy days.

The invention of the waterproof-cloth was the culminating point of Syme's chemical studies and experiments. He was now drawn by the instinct that had made him in his boyhood make those dainty skeletons of frogs and mice "and such small deer," to the study of anatomy. He was fortunate in his teachers in that science. Not only was Dr. Barclay, the professor, an enthusiastic anatomist, with the gift of arousing equal enthusiasm in the breasts of his students, but he had for his chief demonstrator Robert Liston, the famous surgeon of after days. Between Liston and Syme there sprang up a friendship so close that when in the autumn of 1818 there arose a misunderstanding between Liston and Barclay, and the former gave up his post in the professor's class-room and started as an extra-mural lecturer on anatomy, Syme seceded with him, and became in turn his demonstrator. Having himself studied anatomy for only one year, it may be surmised that he was not at first altogether at ease in his new position, especially as among his sixty pupils there were many whose age outreached his own twenty years; but diligence and earnestness, aided by Liston's encouragement, carried him through, and he became a successful demonstrator. After a visit to Paris, where the facilities for operative surgery were then greater than in Britain, he returned to Edinburgh to undertake the class of Systematic Anatomy in the Extra-Mural School, while Liston took up surgery as his subject.

*(To be continued.)*

